# Early acquisition of complex syntax in Mandarin-speaking infants

**DOI:** 10.1038/s41598-025-01096-x

**Published:** 2025-05-17

**Authors:** Jingtao Zhu, Anna Gavarró

**Affiliations:** 1 Departament de Filologia Catalana, Universitat Autònoma de Barcelona, Barcelona, Spain; 2 ClicAsia, Centre d´Estudis Orientals, Barcelona, Spain

**Keywords:** Non-canonical word orders, Mandarin Chinese, Early development, *Ba* construction, agent-first, Grammatical knowledge, Psychology, Human behaviour

## Abstract

Although Mandarin is an S(ubject)V(erb)O(bject) language, other non-canonical sentences with the object marker *ba* are also possible, yet their comprehension in child Mandarin is underexplored. This study uses eye-tracking and the intermodal preferential looking paradigm, as well as the use of pseudo-verbs, to explore how 24 Mandarin infants (mean age: 17.5 months) and 48 adults process these structures. The results of our experiments show that both infants and adults looked longer at the target scenes for the three grammatical sentence types tested: SVO, S*ba*OV and O, S*ba*OV. While comprehension of SVO and S*ba*OV could be achieved with an agent-first parsing strategy, the fact that patient-first O, S*ba*OV constructions were also parsed by infants suggests access to grammatical, language-specific knowledge.

## Introduction

Children at the beginning of their syntactic productions appear to have established at least some of the morphosyntactic features of the languages they are exposed to, for example the head initial/head final character of Verb Phrases (VP); their spontaneous productions attest to this fact (see the early observations of Brown^1^ and subsequent work such as Fan and Song^2^; Sugisaki^3^; Wexler^4^). Looking at more subtle syntactic distinctions, the productions of the children at the two-word stage adhere to word order constraints such as the distribution of finite verbs with respect to negation in a language like French (*Elle roule pas* vs. *Elle pas roule ‘It doesn’t roll’, i.e. a finite verb must precede negation *pas*, not follow it; Pierce^5^). Likewise, children exposed to German place their finite verbs in Verb-second position, just as adults do (Poeppel and Wexler^6^). These findings led to the formulation of the Very Early Parameter Setting hypothesis (Wexler^4^), according to which early productions follow the word order patterns of the target grammar the child is acquiring. In this paper we focus on the development of syntax at an earlier stage, when children have not reached the two-word stage yet. There is a growing number of studies in this area, partly enabled by new experimental methods, such as eye-tracking (Tanenhaus et al.^7^), which allow us to address the question of whether infants are able to parse complex syntactic strings. This technique is based on an early observation by Cooper^8^, according to which listeners rapidly fixate their gaze in images depicting the referents of heard speech upon hearing the word for the displayed object. The intermodal visual paradigm has been successfully used to test children’s linguistic knowledge in online sentence comprehension starting with Hirsh-Pasek and Golinkoff^9^. In a now-classic study, Hirsh-Pasek and Golinkoff^9^ tested 17-month-old children with sentences of the type “*Big Bird is washing Cookie Monster*” to see if they relied on word order to comprehend them. It was found that children direct their gaze to the matching screen (i.e. the one describing Big Bird washing Cookie Monster) rather than the one depicting theta-role reversal – in which the theta roles assigned to the subject and object positions are interchanged, in our example Cookie Monster washing Big Bird. The same results also obtained in Fisher^10^ and Gertner et al.^11^ using pseudo-verbs. Gertner et al.^11^ tested the comprehension of grammatical transitive sentences, such as “*The duck is gorping the bunny*” involving pseudo-verbs with English-speaking toddlers aged 21 months. Sentences were illustrated by two causative scenes with alternated agent and patient. The results showed that toddlers who heard SVO sentences looked significantly longer at the matching SVO video than at the mismatched video, suggesting that they interpreted the argument preceding the pseudo-verb as the agent.

However, since the overwhelming majority of natural languages present the SO order, whereas the OS order is rare (about 3.4%, Dryer^12^), the prevalence of subject-initial word orders could suggest a tendency for young infants to assume that the first NP in a two-NP sentence is the agent of a causative transitive sentence, as postulated by Bever^13^, and, more recently, Chan et al.^14^, Dittmar et al.^15^, and Lidz et al.^16^. Thus it could be the case that infants’ preference for the SVO over the OVS order in Gertner et al.^11^ is just a reflection of this agent bias and not of knowledge of abstract phrase structure.

Lassotta et al.^17^ (see also Lassotta^18^), using eye-tracking techniques and pseudo-verbs, tested 28 French-learning children with a mean age of 22 months with SVO sentences and the so-called Clitic Left Dislocation construction, illustrated in (1). They used theta-role reversal distractors and the children directed their gaze preferably to the target video. They provided the first direct evidence that French infants can assign the target patient-agent-verb interpretation to non-canonical OSV sentences with pseudo-verbs.

1. Le garçon, la fille le dase.
  The.masc boy_i_ the.fem girl Cl.masc_i_ PSEUDO-V.
  The boy, the girl is dasing him.

Thus, by using cognitively undemanding methods such as eye-tracking, we can test infants before the earliest productions of multi-word utterances. We inquire whether the early representation of grammatical knowledge shown previously for French is general in infants even for a language, like Mandarin, without agreement and Case, given the possibility that there are language-specific timetables for the development of complex grammatical structures. We also put to test the agent-first bias by examining the comprehension of non-canonical patient-first constructions, in particular some constructions of Mandarin which are not agent-first. Our hypothesis is that if infants possess abstract grammatical knowledge they will parse the grammatical agent-first sentences, and also the non-canonical patient-first sentences; in contrast, if an agent-first strategy is at play, they will parse agent-first sentences, but they will fail with non-canonical patient-first sentences.

The paper proceeds as follows. In Sect. "The properties of Mandarin Chinese" we sketch the properties of Mandarin Chinese that are relevant for the understanding of the experiments reported. In Sect. "The present study" we present the experimental design, participants and results for a first experiment conducted with infants and adults, and a follow-up experiment administered to adults. We discuss the results in Sect. “Discussion” and draw the conclusions of our study in Sect. “Conclusion”.

## The properties of Mandarin Chinese

Mandarin Chinese is a very suitable candidate to test the acquisition of early word order. It is a language without subject-verb agreement and no morphological Case marking, and these two properties imply that word order is the main source of information as to syntactic structure. In second place, since Chinese is considered a Topic-prominent language (Li & Thompson^19^; Huang^20^), almost any argument is allowed in Topic position, giving rise to a wide range of word orders. This implies that, in investigating the acquisition of syntax, we can reliably assume that in Mandarin comprehension rests on word order and the matching prosody.

The canonical word order in Mandarin is SVO (Sun & Givón^21^), exemplified in (2) (The following abbreviations are used in the examples: BA = ba construction, CL = classifier, fem = feminine, masc = masculine, PERF = perfective aspect, TOP = topic marker.). In these SVO sentences, the subject is prosodically highlighted and, as the initial topic of the discourse, it raises the initial pitch register and allows the following F_0_ to drop progressively (Wang & Xu^22^).

(2) Xiao-tu-zi zhua le xiao-ya-zi.
  little-rabbit catch PERF little-duck.
  The little rabbit caught the duckling.

Under certain circumstances, the object can be moved to preverbal position, leading to the SOV *ba* construction, exemplified in (3a). Additionally, when the shifted object is inanimate, Chinese permits bare SOV sentences like (3b), (In fact, previous work has shown that the bare SOV word order is subject to further restrictions (Hou^23^) and is difficult to process compared to the OSV word order (Li et al.^24^).) with a special intonation and pause between subject and object.

(3) a. Ta ba ping-guo chi le.
  s/he BA apple eat PERF
  S/he ate the apples.
  b. Ta, ping-guo chi le.
  s/he apple eat PERF
  S/he ate the apples.

However, if the shifted object is animate, then SOV obligatorily requires the morpho-syntactic marker *ba* (van Bergen^25^), which is semantically associated with highly transitive, resultative events (Li^26^; Sun^27^; Wang^28^). This was corroborated experimentally in a recent grammaticality judgement task (Yu & Tamaoka^29^) in which the S(animate)-O(animate)-Verb sentences without *ba* were judged unacceptable among native speakers.

For an analysis of the *ba* construction, we refer the reader to Huang et al.^30^, Kuo^31^, Tsao^32^. For the purposes of our study, suffice to say that the post-*ba* NP is contrastive, and therefore it is obligatory as in (4). As any contrastive topic, it may bear a topic marker, such as *ne* in (5).

(4) Xiao-tu-zi ba xiao-ya-zi zhua le.
  little-rabbit BA little-duck catch PERF
  The little rabbit caught the duckling.
(5) Wo ba zhe ge bao ne fang sha-fa shang le, ba na ge bao.
  I BA this CL bag TOP put sofa up PERF, BA this CL bag.
  ne fang yi-zi shang le.
  TOP put chair up PERF
  I have put this bag on the sofa and that bag on the chair.

The predicate in the *ba* construction is also subject to restrictions, as it must be construed as resultative (as illustrated by the presence of resultative *le* in (3a) above); stative and psychological predicates are therefore excluded from the construction. For a recent analysis of the *ba* construction capturing its syntactic and semantic properties, see Sun^27^.

In Mandarin, the object can also appear in a topic position in the left periphery of the clause (see Chen^33^; Huang^20^; Huang et al.^30^; Xu & Langendoen^34^, among others), together with a coindexed resumptive pronoun as post-*ba* NP in preverbal position (6).

(6) Xiao-ya-zi, xiao-tu-zi ba ta zhua le.
  little-duck_i_ little-rabbit BA it_i_ catch PERF
  The duckling, the bunny caught it.

The left-dislocated topic *xiao-ya-zi* ‘the duckling’ is coreferential with the overt pronoun *ta* ‘it’, as an overt object is mandatory in the *ba* construction. Huang et al.^30^ argued that *ta* ‘it’ in (6) is a resumptive pronoun, which accordingly means that (6) has a gap in the comment clause as well. In addition, prosodically there is a pause between the first NP (i.e. the object) and the second NP (i.e. the subject). Note that left-dislocated topic structures as in (6) differ from dangling topic structures, (The dangling topic constructions are also called Chinese-style topic constructions (Chafe^35^), which are rarely seen in other languages (see Pan & Hu^36^; Shi^37^ for the analysis), while the non-dangling topic constructions, the ones tested in our study, are usually considered a common type, existing in English and many other world languages (although with some differences between them).) shown in (7) (taken from Huang^20^), which are gapless topic constructions, i.e. there is neither a syntactic gap nor a resumptive pronoun in the comment clause (Chafe^35^; Chao^38^; Chen^39^; Huang et al.^30^).

(7) Tan-men, wo kan ni, ni kan wo.
  they, I look you you look I
  They look at each other.

While the preposed object of SOV could be interpreted as either Topic (Tsao^40^; Paul^41,42^) or Focus (Ernest & Wang^43^; Tsai^44^), the first NP in the left-dislocated OSV structure can only be interpreted as a topicalized object, and the second NP as the subject of the sentence, and this is especially true when the object bears a [+ human] feature (Li & Wei^45^).

Existing research on the acquisition of the *ba* construction has focused mainly on production, showing that Mandarin-speaking children can produce the *ba* construction at least when they approach their second birthday (Yang & Xiao^46^; Zhu & Gavarró^47^). Relevant examples from CHILDES (MacWhinney^48^) are given in (8) (taken from Zhu & Gavarró^47^).

(8) a. Ba da-hui-lang gan zou le. (Haohao, 1;8)
  BA wolf drive away PERF.
  I drove the wolf away.
  b. Bang wo ba ta na chu-lai. (Liuzonghao, 2;2)
  help I BA it take out.
  Help me to take it out.
  c. Ta ba ping-guo reng diao le. (Marui, 2;8)
  he BA apple throw away PERF
  He threw away the apple.
  d. Ni ba zhe ge na zou, ba ge na chu-lai. (Lianlian, 3;0)
  you BA this CL take away, BA that CL take out
  You take this away, and take that out.

Although topic structures are produced from a very early age, they are not abundant in the spontaneous speech of children and adults. Sentences departing from the canonical SVO make up only 10% of child-directed speech (Yeh^49^). Given the complexity of the *ba* construction, its acquisition is an arduous task for L2 Mandarin Chinese learners (Huang & Yang^50^; Wen^51^), and native-like performance is only found at the advanced level (Wen^51^).

In view of the high frequency of the canonical word order in child-directed speech, one might hypothesize that Mandarin-speaking children might acquire the predominant SVO order early and develop other word order combinations later. This expectation, if true, would seemly go along with the idea of frequency-based accounts of language acquisition (Yang^52,53^). However, to date, there are no studies examining the acquisition of non-canonical word orders of very early Mandarin grammar, particularly on comprehension before age two. For children of age 2 and older, the study by Hsu^54^ investigated the comprehension of SVO and the *ba* construction with and without a subject (i.e. S*ba*OV and *ba*OV) with pseudo-verbs, using the forced-choice pointing paradigm. The results showed that participants performed significantly above chance level for both constructions and there were no comprehension differences among construction types. In particular, the correct interpretation of the subjectless *ba* construction indicates that Mandarin children can rely on the *ba*-marked patient to infer that the agent is null, suggesting that infants by the age of 2 are sensitive to complex syntactic constructions. It is against this background that the current study was conducted.

## The present study

In this study, we investigate the comprehension of both canonical SVO and non canonical S*ba*OV and O, S*ba*OV constructions using eye-tracking measures adopting the intermodal preferential looking paradigm (see Golinkoff et al.^55^ for a review) for both infants and adults, taking as point of reference the experiment of Lassotta et al.^17^ and Lassotta^18^. In O, S*ba*OV the O topic was separated from the comment by a pause. We insert the comma between O and S to indicate this pause in the written language, but note that a pause or intonational change at this position is not obligatorily required in the spoken language with an inanimate object (Xu^56^). However, having two animate NPs followed by the verb without a pause has very poor acceptability among Chinese speakers (Yu & Tamaoka^29^). The experiments reported were approved by the Ethics Committee on Animal and Human Experimentation at the Universitat Autònoma de Barcelona (CEEAH approval number 5071) and all methods were performed in accordance with the relevant guidelines and regulations. Besides, informed consent was obtained from all participants and/or their legal guardian(s).

### Experiment 1: infants

#### Participants

We tested twenty-four typically developing Mandarin infants with a mean age of 17.5 months (*SD* = 2.2, age range: 13 to 21 months, 12 boys and 12 girls). Thirteen additional infants participated in the study, but they were not included in the results due to their poor tracking quality (*n* = 5) or because of the infants’ lack of compliance with the task or their gaze being outside the screen with consequent loss of data (*n* = 8).

The child participants were recruited in Guiyang, China, and had no reported history of speech, hearing or language disorders. Infants’ vocabulary was measured using the Mandarin version of the Communicative Development Inventory (CDI, Hao et al.^57^), which includes an infant checklist (used for infants from 12 to 16 months of age) and a toddler checklist (used for children between 17 and 30 months). The age division into two groups is given by the questionnaire itself; following Hao et al.^57^, for the toddler list parents were only asked to indicate whether their children had ever said the word, thus no comprehension scores are available.

In our study, infants from 13 to 16 months (younger group, *n* = 12) achieved a mean score of production of 9 words (*SD* = 7.1, range from 1 to 22) and a mean score of comprehension of 42 words (*SD* = 27.7, range from 2 to 88). Infants from 17 to 21 months (older group, *n* = 12) achieved a mean score of production of 67 words (*SD* = 74.9, range from 6 to 295). The summary of these measures is shown in Table 1.

**Table 1:** Infants’ vocabulary scores.

Months	Comprehension (words) Mean	Range	Production (words) Mean	Range
13–16	42 (*SD* = 27.7)	2–88	9 (*SD* = 7.1)	1–22
17–21	-	-	67 (*SD* = 74.9)	6-295

Because of the relatively large age range in our study and in order to detect age-related trends (if any), we treat age as a continuous variable.

#### Materials and design

We tested both canonical SVO (9a) and non-canonical sentences involving the *ba* construction (9b, c). Noun phrases referred to highly frequent animals in Chinese story telling (a cow, a dog, a duck, a rabbit, a sheep, and a horse). All the children knew the name of the animals used in the experiment according to their vocabulary checklists.

(9) a. Xiao-tu-zi tuān le xiao-gou.
  little-rabbit PSEUDO-V PERF little-dog
  The bunny V-ed the puppy.
  b. Xiao-tu-zi ba xiao-ya-zi tuān le.
  little-rabbit BA little-duck PSEUDO-V PERF ‘The bunny V-ed the duckling.’
  c. Xiao-ya-zi, xiao-tu-zi ba ta tuān le.
  little-duck little-rabbit BA it PSEUDO-V PERF
  The duckling, the bunny V-ed it.

A total of nine target items (three for each condition) were constructed as listed in Table 2. We used the same pseudo-verb *tuān* ‘to put a colander on someone’s head’ in all conditions. Each sentence was associated to a synchronized pair of videos depicting the puppet characters carrying out actions that are not lexicalized in Mandarin. One video represented the target transitive event, the other depicted the same event involving the same characters with theta-role reversal.

**Table 2:** List of experimental sentences.

SVO	Xiao-tu-zi tuān le xiao-gou. little-rabbit PSEUDO-V PERF little-dog
	Xiao-ya-zi tuān le xiao-ma. little-duck PSEUDOV PERF little-horse
	Xiao-niu tuān le xiao-ma. little-cow PSEUDOV PERF little-horse
S*ba*OV	Xiao-tu-zi ba xiao-ya-zi tuān le. little-rabbit BA little-duck PSEUDO-V PERF
	Xiao-niu ba xiao-ma tuān le. little-cow BA little-horse PSEUDO-V PERF
	Xiao-yang ba xiao-gou tuān le. little-sheep BA little-dog PSEUDO-V PERF
O, S*ba*OV	Xiao-ya-zi, xiao-tu-zi ba ta tuān le. little-duck_i_ little-rabbit BA it_i_ PSEUDO-V PERF
	Xiao-gou, xiao-niu ba ta tuān le. little-dog_i_ little-cow BA it_i_ PSEUDO-V PERF
	Xiao-yang, xiao-ma ba ta tuān le. little-sheep_i_ little-horse BA it_i_ PSEUDO-V PERF

**Fig. 1:**
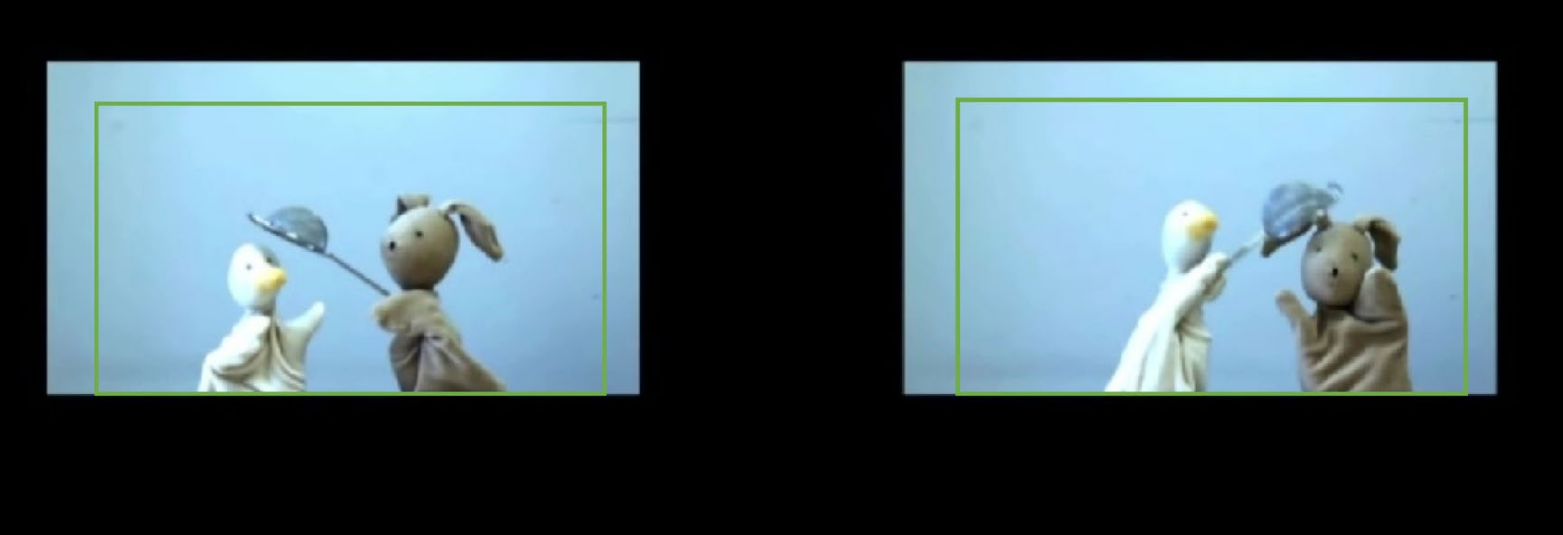
Materials corresponding to (9b) and (9c), taken from Tobii Studio™ (v3.4.8) by the first author. The rectangles indicate the Regions of Interest (ROI) used in our measures.

The materials are illustrated in Fig. 1.

The nine test items were arranged in random order, with the presentation of the target and reverse actions counterbalanced across the left and right sides of the screen.

#### Production of the test stimuli

The test sentences were produced by a female native speaker of Mandarin. She was asked to produce all sentences with target intonation. The recording was conducted in a sound-attenuated recording studio. We used Adobe Premier Pro CC 2017 (v11.0.2) to edit the visual stimuli and Praat (Boersma & Weenink^58^) to record the spoken utterances.

#### Experimental procedure

The participants’ eye movements were recorded using a Tobii Pro X3-120 (with a sampling rate of 120 Hz) interfaced with a portable computer. Tobii Studio™ (v3.4.8) was used as the platform for the recording and analysis of eye gaze data. Spoken utterances were presented to the participants through two external speakers connected to the computer. Each child sat on his or her caregiver’s lap during the whole length of the experiment in order to elicit natural behaviour (Hessel et al.^59^). The distance between the participants’ eyes and the monitor was about 60 cm. The caregivers were asked to close their eyes and listen to music played through headphones during the test trials so as not to guide their children towards any of the videos.

The participants were tested individually. Standard calibration was conducted before the experiment started, where a researcher, blind to the stimuli, observed the participants and clicked the mouse. Participants fixated on a grid of five calibration points in a predetermined order (Although using the 9-point method registers more successful calibration points compared to the 5-point (Zeng et al.^60^), considering the young age of the participants, we opted for a 5-point calibration.). After having arrived at a satisfactory level of validity, the recording of eye movements took place from the onset of the training session and throughout. All that was expected of the participants is that they watch the video.

During the training session, first the participants went through a character-identification phase; all the puppets were presented once, either on the left or on the right side of the screen (e.g. *Bao-bao kuai kan*,* shei zai na-r? O*,* shi xiao-tu-zi*. ‘Look, who’s here? It’s the bunny’), while the other screen remained blank (6s). In the second phase, the participants were introduced to the simultaneous presentation of two videos, which showed two different animals at the same time while the recorded audio asked them whether they saw one of them (e.g. *Bao-bao nikan*,* kan-dao xiao-tu-zi le ma? Xiao-tu-zi zai na-li ya?* ‘Look, do you see the bunny? Where is the bunny?’). The third phase familiarized the participants with the novel action. The novel action was presented once on the left, once on the right of the screen and it was presented only in neutral frames such as *Bao-bao kuai kan*,* fa-sheng le shen-me?* ‘Look, what happened?’ so that later understanding of the test sentences cannot be attributed to lexical learning during the training phase (see Ambridge & Lieven^61^; Franck et al.^62^ for discussion).

**Fig. 2:**
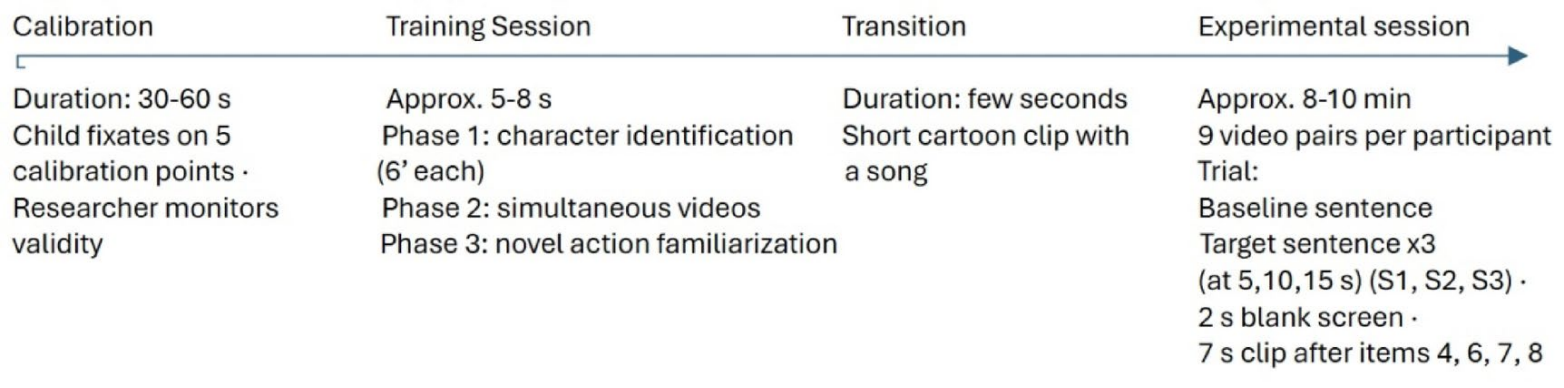
Timeline of the experiment.

After the training session and a short transition cartoon with a song, the experimental session started. In the experimental session, all videos (= 9 pairs) started with a sentence drawing the attention of the participant (e.g. *Bao-bao kuai kan*,* fa-sheng le shen-me?* ‘Look, what happened?’) as baseline, and then the experimental sentences were played three times. The recording of gazing time took place in four windows: the baseline and the three consecutive exposures to the target sentence starting at 5, 10, 15 s. Exposure to the baseline and to the (repeated) target sentence determined the time windows for video segmentation. A blank screen (2s) appeared between experimental items, and after items 4, 6, 7 and 8 a 7-second-long clip of the Teletubbies landscape was shown to keep the child’s attention. The timing of the stimuli the infants received is summarized in Fig. 2.

The whole session lasted about 20 min. After the test session, the experimenter asked the infants’ caregivers to fill out the Chinese version of CDI (Hao et al.^57^).

#### Data treatment and analysis

Participants were excluded if there was more than 45% track loss from the first trial in the testing phase after training, namely only infants with more than 55% of detected signal in each of the four windows of analysis were taken into account (mean gaze sample = 68.25%, *SD* = 10.46 (The percentage of away looks was 29.8% for the SVO condition, 31.29% for the S*ba*OV condition, and 33.15% for the OS*ba*OV condition. A repeated measures ANOVA was performed to compare the percentage of away looks across conditions. Results showed that the differences among conditions in terms of away looks were not statistically significant (*F*(2, 66) = 0.58, *p* =.56, η_p_^2^ =0.017)).This criterion was applied given the young age of the participants, which advises against a stricter criterion, and following the standard procedures in the literature (e.g. Franck et al.^62^; Gavarró et al.^63^); as a result, eight trials were excluded for infants, as described in 3.1.1.

In analyzing the eye movement data, we first categorically partitioned the data from the onset into four temporal windows: the baseline window (BS) and three consecutive exposures to the target events (S1, S2, S3), starting at 5, 10, 15 s respectively. Then participants’ fixations were coded in two scenes containing the target and non-target (i.e. reverse) events.

Mean looking times and proportion of fixation (calculated over the total looking time to the target and non-target videos) on each ROI in a specific temporal window were calculated. To provide an overview of the eye movement data, statistical analysis involved bivaried paired Student *t* tests for means’ comparisons and linear mixed-effects models for analysis of fixation proportions (see Zhan^64^ for some discussion on this topic) using the lme4 (v1.1-12) package (Bates et al.^65^) from R (v3.2.5, R Development Core Team^66^), and we used the Wald test to compute *p*-values for each fixed effect and the interactions. For multiple comparisons, we used the Bonferroni correction. We fit the data for each condition (SVO, S*ba*OV and O, S*ba*OV) separately, focusing on the gaze duration toward the two critical scenes (target and reverse scenes) in each condition. According to the principle of parsimony (Vandekerckhove et al.^67^), we accepted the simplified model if this model could explain the same variance as the full model. In the full model, the fixed effects included Scene (target versus reverse), Window (baseline BS, first S1, second S2 and third S3) and their interactions; the random effects included items and subjects, where both their intercepts and slopes were allowed to vary among all the fixed effects. To compare the fit of two models, we used the anova() function in R. The final model treated the interactions between Scene (target versus reverse) and Window (baseline BS, first S1, second S2 and third S3) as fixed effects, with random intercept and slope for participants and items (to be found below, at the end of Tables 4-6 and 8-10 respectively). Since fixation proportions follow a multinomial distribution rather than a normal distribution, they should be transformed to unbounded variables with the empirical logit formula when using linear models (Barr^68^). Following Barr^68^, fixation proportions were transformed using In [(*y* + 0.5)/(*n* -*y* + 0.5)], where *y* is the number of fixations on each scene during a particular temporal window, and *n* is the total number of fixations in that window. Finally, we explored the effect of age (as a continuous variable, in days) and vocabulary (as a continuous variable, number of words produced (In the CDI, as mentioned, the only measure of vocabulary growth given for younger and older infants is word production, not comprehension; therefore, that is the measure that is used in the comparisons.)) using generalised linear models with the proportion of looking time to the target scene as dependent variable.

#### Predictions

If sentence comprehension in infancy is guided by adult-like abstract syntactic knowledge, we expect infants to show a preference for the scene with the first NP being the agent in the canonical SVO and non-canonical S*ba*OV conditions, whereas, in the non-canonical O, S*ba*OV, a preference for the scene with the first NP being the patient is expected.

By contrast, if children’s sentence comprehension is guided by heuristics like assigning the agent role to the first noun, then a preference for the scene with the first NP being interpreted as the agent is predicted in the three O, S*ba*OV, SVO and S*ba*OV conditions.

We do not make any specific predictions as to when identification of the target video will take place; infants tested in this paradigm standardly hear three consecutive presentations of the target sentence, and we are not aiming at establishing at which exact point they will be able to fixate on the target; rather our goal is to determine whether they are able to identify the target video or not.

#### Results

The results of mean looking time appear in Table 3.

**Table 3:** Mean looking times (in Ms, *SD* in parentheses) across the four critical Temporal windows in three conditions comparing target and reverse (infants) (Recall that the target and reverse videos are not the same across conditions.).

	SVO		SbaOV		O, SbaOV	
	Target	Reverse	Target	Reverse	Target	Reverse
BS	1299(589)	1410(820)	1931(852)	1659(746)	1394(665)	1163(715)
S1	1511(916)	1451(853)	2273(1103)	1829(957)	**1867(986)** ^**^	**1289(785)**
S2	**1660(837)** ^*^	**1139(890)**	**1944(1193)** ^*^	**1396(933)**	**1888(1102)** ^*^	**1323(868)**
S3	**1523(865)** ^***^	**1026(628)**	1595(1079)	1900(1204)	1364(1060)	1290(825)

^*^p < .05, ^**^p < .01, ^***^p < .001 (in bold).

These results show that, in the SVO condition, infants looked significantly longer at the target video than at the reverse video during the second (t(23) = 2.40, *p* = .025, Cohen’s *d* = 0.60, *p* corrected = 0.042) and the third exposures to the test sentence (t(23) = 3.99, *p* = .001, Cohen’s *d* = 0.66, *p* corrected = 0.028), reflecting their target SVO interpretation rather than OVS. No significant difference was found in the baseline window, nor during the first presentation of the sentence. In the non-canonical S*ba*OV condition, infants still preferred looking at the target scene, with the first NP corresponding to the agent, during the second presentation of the sentence (t(23) = 2.19, *p* = .039, Cohen’s *d* = 0.51, *p* corrected = 0.049). However, in the condition with object topicalisation O, S*ba*OV, infants disfavoured the scene with the first NP corresponding to the agent and preferred looking at the opposite scene instead, i.e. the scene in which the first NP corresponded to the patient; this happened from the first presentation of the sentence (t(23) = 3.35, *p* = .003, Cohen’s *d* = 0.65, *p* corrected = 0.030) and the effect marginally lasted until the end of the second exposure to the test sentence (t(23) = 2.08, *p* = .049, Cohen’s *d* = 0.57, *p* corrected = 0.054).

**Fig. 3:**
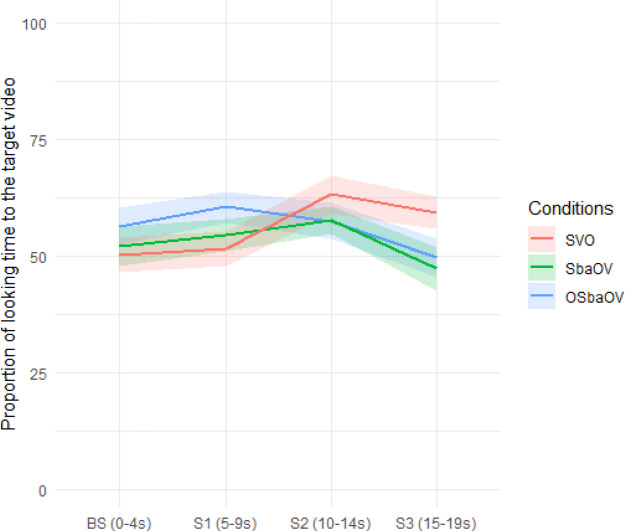
Proportion of looking time to the target video during the four temporal windows in the three conditions with standard error shading (infants).

Proportions of looking time to the target video (calculated over the total looking time to the target and non-target videos) in the four windows is graphically represented in Fig. 3.

Wilcoxon matched-pair analysis of proportions of looking time to the target video in the canonical SVO sentences indicated that infants showed a significantly above chance effect (defined as 50%) during the second (*Z* = -2.80, *p* = .005, *r* = .57) and the third exposures to the test sentence (*Z* = -2.53, *p* = .011, *r* = .52). In the non-canonical S*ba*OV condition, infants showed above chance gazing to target during the second presentation of the sentence (*Z* = -2.34, *p* = .019, *r* = .49), and in the non-canonical O, S*ba*OV condition they showed significantly above chance gazing to target during the first presentation of the sentence (*Z* = -2.77, *p* = .006, *r* = .57). Looking times in the other windows were no different from chance.

Besides, linear mixed-effects models were computed, and proportions were transformed with the empirical logit formula mentioned in Sect. 3.1.5. Table 4 summarises the results of the model for the infant data in the SVO condition (S1, S2 and S3 are correlated, given that they correspond to the response to the same sentence repeated three times. If instead of analyzing each sentence presentation separately to compare it to the baseline, we group S1, S2 and S3 (the mixed effects structure we already had, with participant and item intercepts), we can consider the difference of S1-S2-S3 pooled together vs. the baseline. When we did that, for SVO the pooled analysis showed an overall effect (β = 0.069, t = 1.53, *p* = .013), which means the effect of the target (compared to baseline) is consistent in this condition; for S*ba*OV the difference between the target condition and the baseline was not statistically significant (β = 0.011, t = 0.27, *p* = .079); the pooled results showed a marginally significant difference between the target condition and the baseline in O, S*ba*OV condition (β = 0.021, t = 0.67, *p* = .051).). During the second and the third exposures to the target sentence, hearing SVO sentences caused the infants to look significantly longer at the target scene than at the reverse scene (β = 0.36, *p* = .01 for the second exposure and β = 0.29, *p* = .04 for the third exposure to the target sentence). The significant negative coefficient for the interaction between reverse scene and second and third exposures to the target sentence indicates that the probability of looking toward the reverse scene (i.e. the scene with the first NP as patient) decreased over time after hearing the SVO sentences.

**Table 4:** Fixed effects from the best-fitting model of probability of looks to the target and reverse scenes in the SVO condition (empirical logit transformed), infants.

Condition	Fixed effects	Estimate	SE	t Value
	(Intercept)	-0.022	0.095	-0.238
	Scene (reverse)	0.045	0.150	0.303
	S1	0.050	0.139	0.362
SVO	S2	0.361	0.139	2.596 ^*^
	S3	0.285	0.139	2.050 ^*^
	Scene(reverse): S1	-0.100	0.191	-0.527
	Scene(reverse): S2	-0.722	0.191	-3.780 ^***^
	Scene(reverse): S3	-0.570	0.191	-2.986 ^**^

Formula in R: Proportion ~ Scene*Window+(1 + Scene + Window|Subject)+ (1 + Scene + Window|Items).

As shown in Table 5, during the second exposure, hearing S*ba*OV sentences caused the infants to look significantly longer at the target scene than at the reverse scene (β = 0.16, *p* =. 048). The negative coefficient for the interaction indicates that the probability of looking at the reverse scene decreased over time when the infants heard the S*ba*OV sentences during the second presentation.

**Table 5:** Fixed effects from the best-fitting model of probability of looks to the target and reverse scenes in the S*ba*OV condition (empirical logit transformed), infants.

Condition	Fixed effects	Estimate	SE	t Value
	(Intercept)	0.093	0.120	0.777
	Scene (reverse)	-0.186	0.189	-0.985
	S1	0.068	0.147	0.460
S*ba*OV	S2	0.162	0.147	1.102^*^
	S3	-0.207	0.147	-1.043
	Scene(reverse): S1	-0.136	0.208	-0.651
	Scene(reverse): S2	-0.325	0.208	-1.558^*^
	Scene(reverse): S3	0.413	0.208	1.084

Formula in R: Proportion ~ Scene*Window+(1 + Scene + Window|Subject)+ (1 + Scene + Window|Items).

As shown in Table 6, in the O, S*ba*OV condition, the positive coefficient for the significant main effect of the first and the second exposures to the test sentence reflects the fact that the infants looked significantly longer at the target scene compared to the baseline window when they heard the O, S*ba*OV constructions. The negative coefficient for the interaction confirms that, for infants, the probability of looking at the reverse scene decreased over time after hearing the first and the second presentation of the test O, S*ba*OV sentences.

**Table 6:** Fixed effects from the best-fitting model of probability of looks to the target and reverse scenes in the O, S*ba*OV condition (empirical logit transformed), infants.

Condition	Fixed effects	Estimate	SE	t Value
	(Intercept)	0.165	0.104	1.582
	Scene (reverse)	-0.231	0.197	-1.171
	S1	0.473	0.199	2.376 ^*^
O, S*ba*OV	S2	0.494	0.208	2.500 ^*^
	S3	-0.029	0.270	-0.141
	Scene(reverse): S1	-0.347	0.270	-1.282^*^
	Scene(reverse): S2	-0.334	0.270	-1.235^*^
	Scene(reverse): S3	0.156	0.270	0.579

Formula in R: Proportion ~ Scene*Window+(1 + Scene + Window|Subject)+ (1 + Scene + Window|Items).

We also explored the main effect of sentence condition and fixation to the areas with agent-first (i.e. the target scene in SVO and S*ba*OV conditions, the reverse scene in O, S*ba*OV condition). We found a significant interaction between them. Hearing the SVO sentences caused the infants to look significantly longer at the scene with the first NP as agent (β = 0.65, t = 4.60, *p* < .001), the same happened when they heard the S*ba*OV sentences (β = 0.55, t = 3.93, *p* < .001). However, infants exhibited the opposite eye movement pattern when they heard the O, S*ba*OV sentences (β = − 0.19, t = -2.75, *p* = .006), that is, hearing O, S*ba*OV triggered more fixations to the scene with the first NP as patient. The eye patterns provide evidence that they were able to identify the thematic roles encoded in word orders rapidly and effectively during real-time sentence comprehension. Finally, generalized linear models with the proportion of looking time to the target scene as dependent variable were conducted to compare the main effect of age, vocabulary and their interaction. The age was coded in days, the vocabulary was coded in number of words produced. The results revealed that there was no main effect of age group (β = 0.01, t = 1.08, *p* = .28) or vocabulary size (β = 0.003, t = 0.57, *p* = .56). Besides, the interaction between age and vocabulary size was not significant either (β = − 0.002, t = − 0.47, *p* = .64). To summarize, infants showed the gazing patterns in (10):

(10) a. SVO – Looks at video with S-as-agent.
  b. S*ba*OV – Looks at video with S-as-agent.
  c. O, S*ba*OV – Looks at video with S-as-agent.

### Experiment 2: adult

#### Method

Participants- Twenty-four naïve Mandarin-speaking adults (mean age 28, *SD* = 6.05, age range: 24 to 53 years old) participated in the experiment. They were recruited in Guiyang and the city of Barcelona.

Materials, procedure and data analyses – We adopted the same materials and analyses used for infants. Prior to the task, participants were not given any instructions other than to look at the video. For the adult participants, the detected signal required was more than 75% and no participants were excluded.

#### Results

For adults, the results of mean looking time (see Table 7) show that, except for the baseline window, adults looked significantly longer at the target video than at the reverse video in the canonical SVO condition and non-canonical S*ba*OV condition during the first, the second and the third presentation of the sentence (all *p’*s < 0.001, all *p* corrected < 0.001). In the O, S*ba*OV condition, adults showed no preference for the target video during the first exposure to the test sentence, although they identified the target event during the second (t(23) = 4.78, *p* < .001, Cohen’s *d* = 1.72, *p* corrected < 0.001) and the third presentation of the sentence (t(23) = 4.38, *p* < .001, Cohen’s *d* = 1.63, *p* corrected < 0.001).

**Table 7:** Mean looking times (in Ms, *SD* in parentheses) across the four critical Temporal windows in three conditions comparing target and reverse (adults).

	SVO		SbaOV		O, SbaOV	
	Target	Reverse	Target	Reverse	Target	Reverse
BS	2047(1213)	2599 (1481)	2656(1367)	2673 (1314)	2533(1621)	2403 (959)
S1	**4277(1478)** ^***^	**1881** **(1105)**	**4236(1385)** ^***^	**1831** **(1088)**	3221(1437)	2797 (1380)
S2	**5186(1708)** ^***^	**825** **(1265)**	**5814(1693)** ^***^	**830** **(1042)**	**4856(1794)** ^***^	**1697** **(1864)**
S3	**5315(1715)** ^***^	**905** **(1373)**	**5806(1704)** ^***^	**593** **(785)**	**4600(1602)** ^***^	**1747** **(1876)**

^***^p < .001 (in bold).

**Fig. 4:**
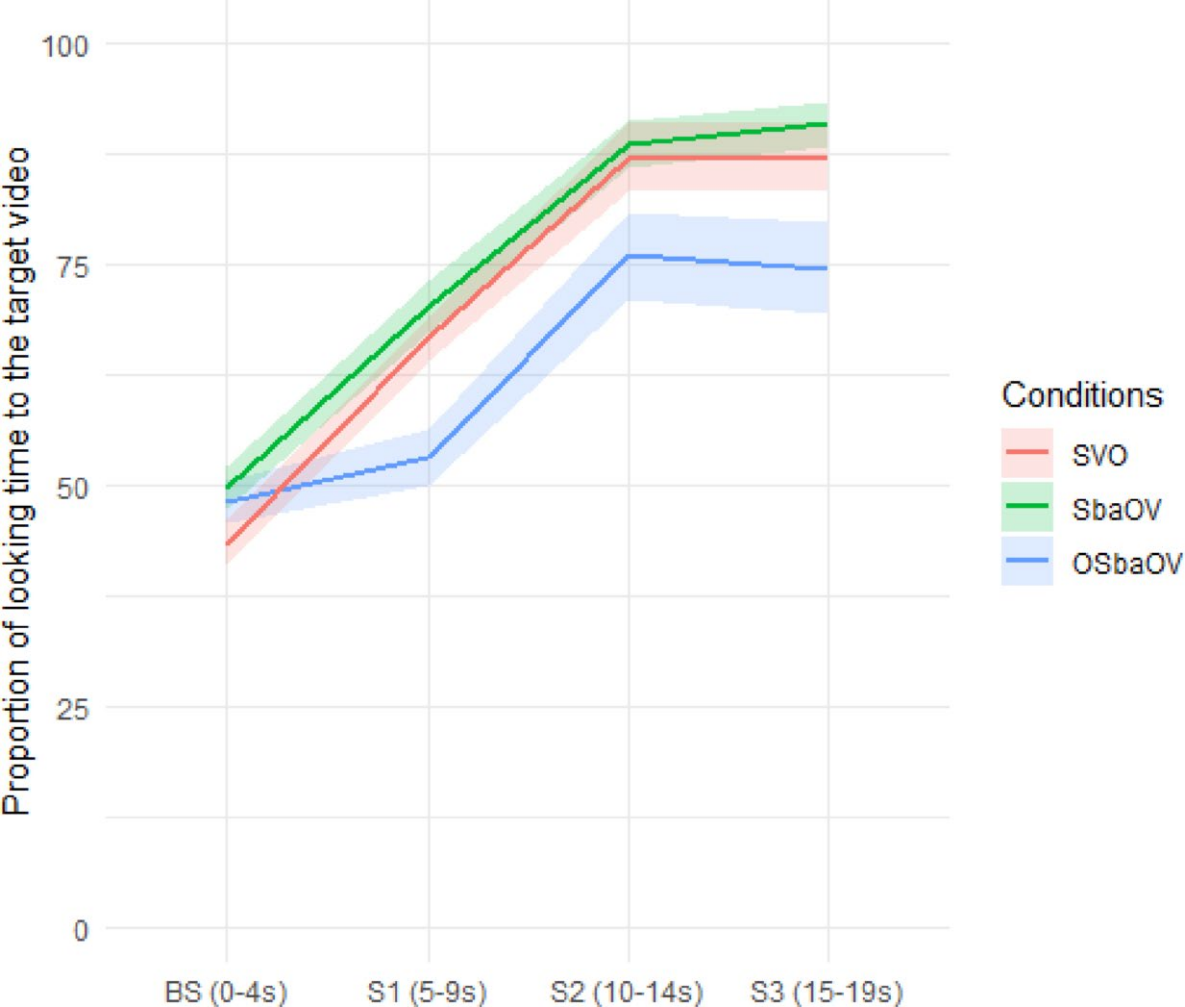
Proportion of looking time to the target video during the four windows in the three conditions and standard error shading (adults).

The proportion of looking time to the target scene is represented in Fig. 4.

Wilcoxon matched-pair analysis of proportions of looking time to the target video indicated that, in both SVO and S*ba*OV conditions, adults showed a significantly above chance effect (defined as 50%) for all windows (all *p’*s < 0.001) except for the baseline. In the O, S*ba*OV condition, except for the baseline and the first exposure to the test sentence, adults also showed significantly above chance looks to the target video for all windows (all *p’*s < 0.001).

We assessed the gaze patterns of adults using linear mixed-effects models, and proportions were transformed with the empirical logit formula mentioned in Sect. 3.1.5. The negative coefficient for the interaction between the reverse scene and window (see Tables 8 and 9) indicates that, in both SVO and S*ba*OV conditions, the probability of looking at the reverse scene decreased after hearing the test sentences.

**Table 8:** Fixed effects from the best-fitting model of probability of looks to the target and reverse scenes in the SVO condition (empirical logit transformed), adults.

Condition	Fixed effects	Estimate	SE	t Value
	(Intercept)	-0.233	0.157	-1.490
	Scene (reverse)	0.467	0.222	2.107
	S1	0.855	0.222	3.855 ^***^
SVO	S2	1.990	0.222	8.972 ^***^
	S3	1.999	0.222	9.013 ^***^
	Scene(reverse): S1	-1.710	0.314	-5.452 ^***^
	Scene(reverse): S2	-3.980	0.314	-12.688 ^***^
	Scene(reverse): S3	-3.998	0.314	-12.747 ^***^

Formula in R: Proportion ~ Scene*Window+(1 + Scene + Window|Subject)+ (1 + Scene + Window|Items).

**Table 9:** Fixed effects from the best-fitting model of probability of looks to the target and reverse scenes in the S*ba*OV condition (empirical logit transformed), adults.

Condition	Fixed effects	Estimate	SE	t Value
	(Intercept)	-0.017	0.131	-0.129
	Scene (reverse)	0.034	0.186	0.182
	S1	0.802	0.186	4.312 ^***^
S*ba*OV	S2	1.789	0.186	9.621 ^***^
	S3	1.938	0.186	10.425 ^***^
	Scene(reverse): S1	-1.603	0.263	-6.079 ^***^
	Scene(reverse): S2	-3.578	0.263	-13.606 ^***^
	Scene(reverse): S3	-3.877	0.263	-14.744 ^***^

Formula in R: Proportion ~ Scene*Window+(1 + Scene + Window|Subject)+ (1 + Scene + Window|Items).

In the O, S*ba*OV condition, there was no main effect of Scene when they heard the sentences for the first time (see Table 10), which means that their interpretation did not significantly differ from that of the baseline window. In the subsequent exposures, they interpreted the first noun as patient efficiently.

**Table 10:** Fixed effects from the best-fitting model of probability of looks to the target and reverse scenes in the O, S*ba*OV condition (empirical logit transformed), adults.

Condition	Fixed effects	Estimate	SE	t Value
	(Intercept)	-0.046	0.183	-0.253
	Scene (reverse)	0.093	0.259	0.357
	S1	0.158	0.259	0.609
O, S*ba*OV	S2	1.253	0.259	4.837 ^***^
	S3	1.210	0.259	4.669 ^***^
	Scene(reverse): S1	-0.316	0.366	-0.861
	Scene(reverse): S2	-2.506	0.366	-6.840 ^***^
	Scene(reverse): S3	-2.420	0.366	-6.604 ^***^

Formula in R: Proportion ~ Scene*Window+(1 + Scene + Window|Subject)+ (1 + Scene + WindowItems).

Surprisingly, adults did not identify the target scene in the O, S*ba*OV condition in the first presentation of the sentence, which can be described as a latency. This means that, unlike infants, as shown in Fig. 3, adults took longer to look at the matching scene, although they identified the target event from the second presentation of the sentence. We hypothesized that this might be a consequence of the fact that the baseline sentence (e.g. ‘*Look*,* what happened*?’) cannot be felicitously answered with a topicalized structure, and this may cause confusion in adults. If this is on the right track, by adding a more restrictive context, namely a narrow focus context (e.g. *Baobao ikan*,* xiao-ya-zi fa-sheng le shen-me?* ‘Look, what happened to the bunny?’), we would predict that adults would improve their performance. We conducted a follow-up experiment to put this prediction to test.

### Follow-up experiment

#### Method

Twenty-five Mandarin-speaking adults (mean age = 29, *SD* = 4.8, age range: 22 to 45 years) participated in this study; all were recruited in the city of Barcelona. One participant was removed due to technical issues that prevented the completion the task; in the end, twenty-four participants were included in the study. The test sentences contained both SVO with neutral context (i.e. *Baobao nikan*,* fa-sheng le shen-me?* ‘Look, what happened?’) and O, S*ba*OV with appropriate contexts for topicalization (e.g. *Baobao nikan*,* xiao-ya-zi fa-sheng le shen-me?* ‘Look, what happened to the bunny?’) as baseline sentences. There were 6 trials in total (3 for each condition). The procedure was the same as in the previous experiments.

#### Results

**Fig. 5:**
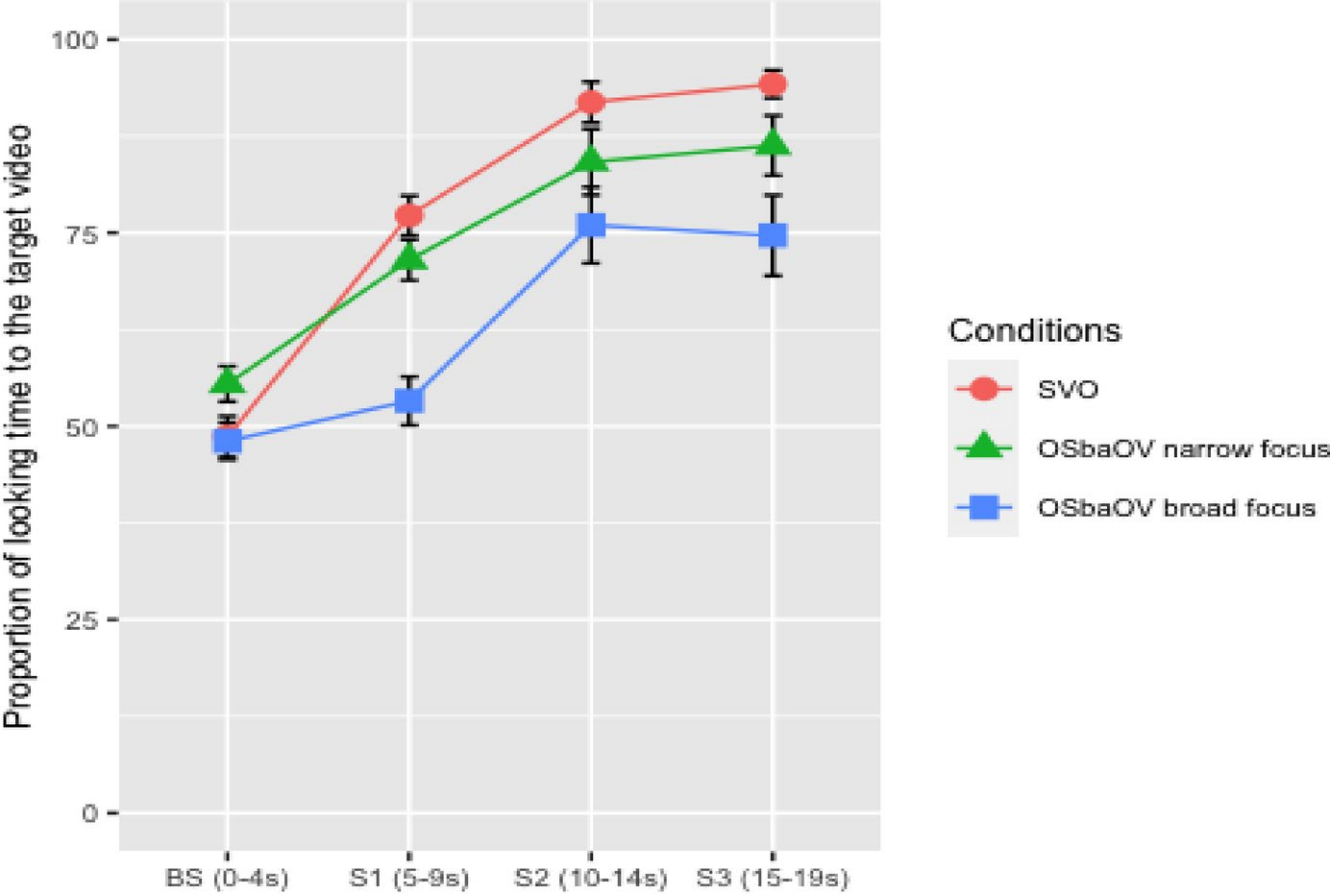
Proportion of looking time to the target video in the four critical temporal windows in SVO and O, S*ba*OV conditions in broad focus context (experiment 2) and O, S*ba*OV in narrow focus context (follow-up experiment) (adults).

The results (see Fig. 5) corroborate our conjecture. Mixed models on proportion of fixation reveal that, in the SVO condition, there was a significant effect of Scene (target vs. reverse) after the first (β = 2.08, t = 12.20, *p* < .001), the second (β = 3.77, t = 22.07, *p* < .001) and the third presentation of the sentence (β = 3.95, t = 23.13, *p* < .001), with a significantly higher proportion of looks to the target agent-first scene. In the O, S*ba*OV condition, there was a significant effect of Scene (target vs. reverse) after the first (β = 1.16, t = 4.04, *p* < .001), the second (β = 2.49, t = 8.70, *p* < .001) and the third presentation of the sentence (β = 2.74, t = 9.56, *p* < .001), with a significantly higher proportion of looks to the target patient-first scene. No latency was found in the first presentation of the sentence, contrary to what was found in the previous experiment.

## Discussion

In the present study, we sought to investigate whether infants exposed to Mandarin are able to parse sentences with canonical and non-canonical word order patterns. Using the intermodal preferential looking paradigm, we found that infants, as early as 17.5 months, can correctly assign the agent role to the first NP in both canonical SVO and non-canonical S*ba*OV constructions; on the other hand, in the O, S*ba*OV condition, infants interpreted the first NP as patient even during the first exposure to the sentence, reflecting their rapid fixation to the target interpretation. Performance with SVO and S*ba*OV is consistent with an agent-first conceptual bias, while with O, S*ba*OV an agent-first strategy would predict systematic miscomprehension. We consider the underpinnings of the analysis in terms of an agent-first strategy, and those of an analysis of performance driven by abstract grammatical knowledge for our Mandarin constructions, and also take into consideration other results available in the literature on Mandarin and other languages. Note that the claim is not that there is no such first-NP-as-agent bias, since the results obtained here do not preclude in any case the existence of such a strategy, instead, the question is whether, even if infants begin with a preference for agent, before they enter the two-word stage they are able to override this preference and know at least some of the syntactic properties of the language they are exposed to. An agent-first strategy cannot be excluded at an earlier stage, whether it is an unborn strategy or a bias that emerges with experience, as suggested by Garcia and Kidd^69^.

The predictions of the agent-first strategy and the grammar-driven parsing for SVO are identical. To tease the two proposals apart, we turn to the other two sentence types in our experiment: S*ba*OV and O, S*ba*OV. In our results, S*ba*OV was comprehended. This result is in contrast with a result from a previous experiment (Zhu et al.^70^, replicating Franck et al.^62^), which showed that children performed at random when they heard an ungrammatical SOV structure without *ba* (exemplified in (11)).

(11) *Xiao-tu-zi xiao-ya-zi nuí le.
  little-rabbit little-duck PSEUDO-V PERF.

**Fig. 6:**
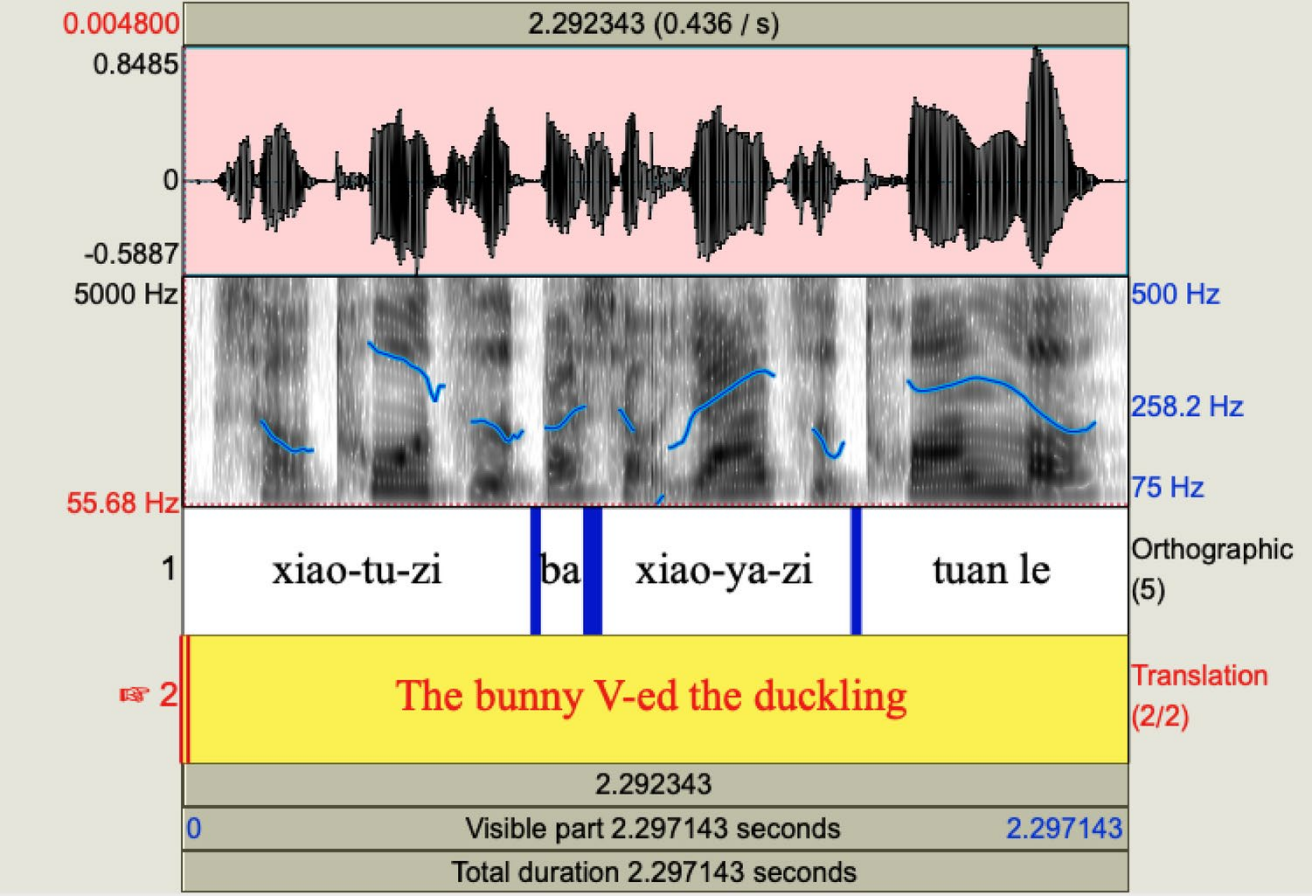
Intonational pattern of the test S*ba*OV sentence *Xiao-tu-zi ba xiao-ya-zi tuān le*.

The infants’ comprehension of grammatical SVO sentences, in contrast with random behaviour with ungrammatical SOV sentences in Mandarin with pseudo-verbs, led the authors to conclude that infants at 17 months recognise SVO as a well-formed structure of their native language, while they do not parse sequences that are alien to their grammatical system. Taking the present results into account, this would indicate that infants exposed to Mandarin are sensitive to the presence of morphosyntactic marker *ba* together with the prosodic properties of the sentences from 17.5 months and they can use this knowledge to parse a sentence. As observed by an anonymous reviewer, our results do not allow us to tease apart the contribution of the intonational contour (given in Fig. 6) and the presence of the morphosyntactic marker *ba*, which cooccur in well-formed sentences. However, the intonational pattern by itself is possible with other topicalization structures and, therefore, does not allow for the identification of object raising on its own.

**Fig. 7:**
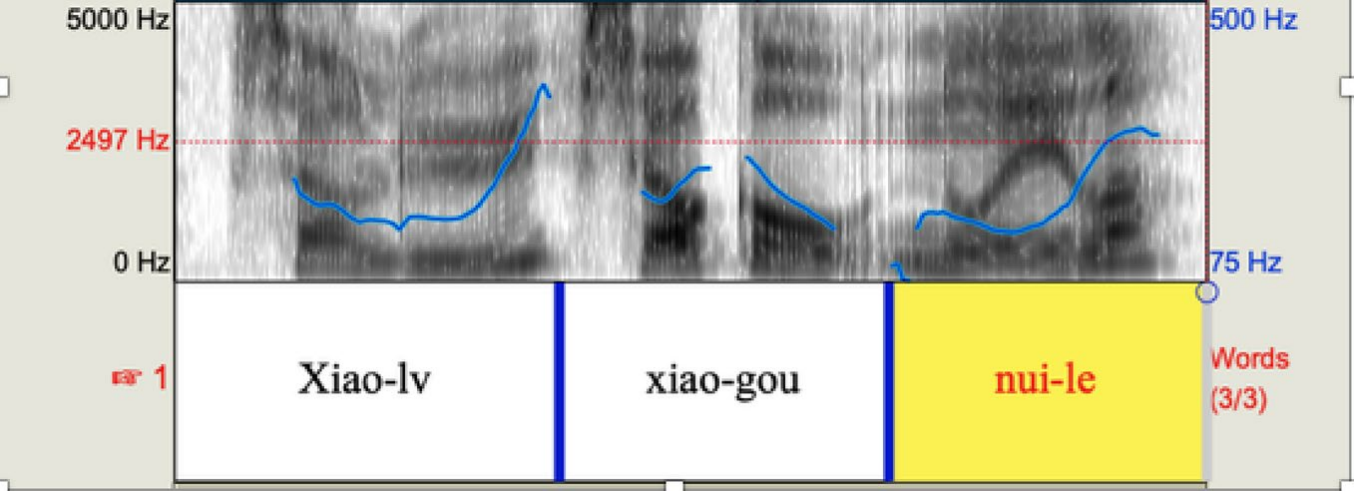
Intonational pattern of the ungrammatical sentence *Xiao-lv xiao-gou nui-le* (from Zhu et al.^70^: pp.67).

A comparison between the study of Zhu et al.^70^ and the study here cannot be performed directly for a reason relating to the experimental design. In Zhu et al.^70^, infants saw two simultaneous videos, one illustrating a transitive event and another describing a reflexive event, with both animals performing the same action, not just one of them as in the present study. In the results of Zhu et al.^70^, infants showed no preference in the SOV condition, while in the study here they directed their gaze to the target video in the S*ba*OV condition. The question is whether the difference in the materials of the two experiments can be a source of the difference in gazing pattern. This seems unlikely given the fact that, as argued by Yang et al.^71^, reversibility of NPs complicates the processing task, and may lead children to take longer to compare two videos to give a response. For that reason, it seems dubious that having theta role reversal distractors would have led to improvement in performance, as is the case. Therefore, the differences between Zhu et al.^70^ and the present study are not likely to contribute to the success of infants here. For this reason, we argue that the difference between performance in SOV and S*ba*OV is genuine and not artifactual. The two sentences, ungrammatical SOV and grammatical S*ba*OV differ by the presence of *ba* and also by their prosody. Compare the intonational contour of the S*ba*OV sentences in our experiment above and that of SOV in Zhu et al.^70^ in Fig. 7.

In previous studies it has been found that children at 25 months of age can use abstract grammatical features, like grammatical gender or aspect, to facilitate sentence comprehension and this sensitivity could be present even much earlier (see Dye et al.^72^ for a review). It is also known that prosody may help identify word order patterns (Bernard & Gervain^73^; Christophe et al.^74^; Gervain & Werker^75^). Given that the morphosyntactic marker *ba* and the grammatical intonation for this sentence type cooccur in the experimental items, the observed effect can result from either one of the factors independently or from their combination. For this condition it is not possible to tease syntax and prosody apart since both are necessary for grammaticality.

Crucially, the agent-first strategy could account for the performance with S*ba*OV here, but not for the performance with SOV in Zhu et al.^70^, as in that study SOV was not comprehended, even though the agent appeared in the first position. This is also the conclusion reached by Franck et al.^62^ for French, another SVO language, and by Gavarró et al.^63^ for Hindi-Urdu, an OV language. In these studies it is argued that performance is consistent with grammar-driven parsing, but not with an agent-first strategy.

Let us now turn to O, S*ba*OV as in (9c) (repeated here for convenience as (12)), which presents the object in initial position and has special prosodic features.

(12) Xiao-ya-zi, xiao-tu-zi ba ta tuān le.
  little-duck little-rabbit BA it PSEUDO-V PERF
  The duckling, the bunny V-ed it.

**Fig. 8:**
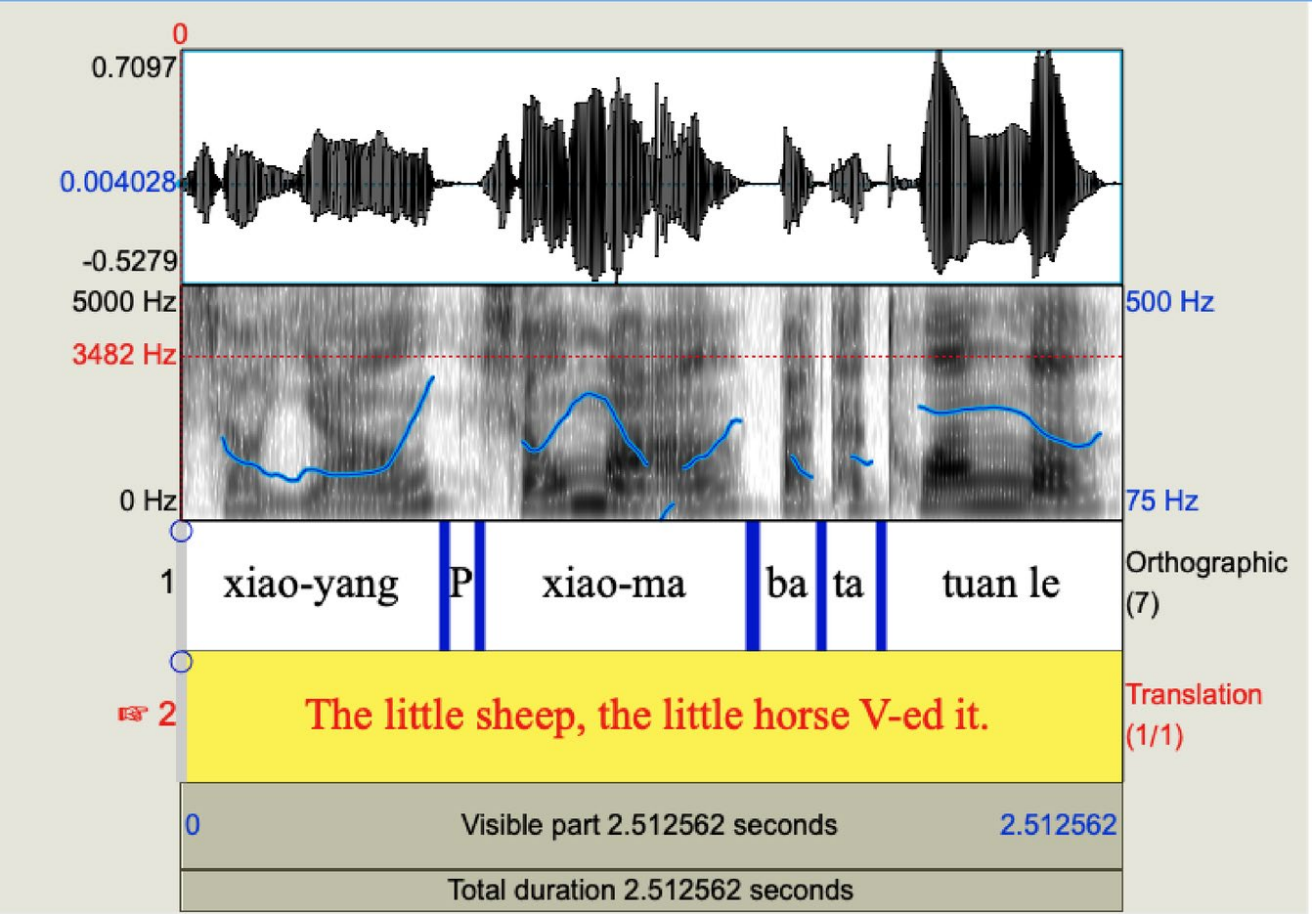
Intonational pattern of the test O, S*ba*OV sentence *Xiao-yang*,* xiao-ma ba ta tuān le.* P represents pause.

O, S*ba*OV is the only construction tested here that requires a pause between the first NP (i.e. the topicalized object) and the second one (i.e. the subject). In our study, the average duration of the pause in the O, S*ba*OV construction between the first NP (the object) and the second NP (the subject) was 373 ms; there was also a pause between the second NP (the subject) and *ba* which had a mean duration of 292ms; see Fig. 8, with the intonational pattern of one of the O, S*ba*OV experimental items. This corresponds to a standard production of (12), although production with a shorter pause after O would also be possible (a pause remains necessary, given that O is animate). Note, moreover, that the same prosodic contour and pause are found with subject topicalizations and, therefore, prosody on its own would not allow the identification of this experimental condition as an object topicalization.

The pause between the object and the subject in O, S*ba*OV structures in our experimental items places the first NP into a separate intonational phrase (in the sense of Nespor & Vogel^76^). The importance of prosodic marking in early parsing is highlighted by the work of Marchetto and Bonatti^77^ showing that 200 ms pauses favour generalization of morphosyntactic regularity to new items in 12-month-olds infants, as word boundaries are made apparent by the pause. It is generally agreed that a pause is the most reliable indicator of topichood in Chinese (Chao^38^). Furthermore, it has been shown that infants and young children are quite sensitive to prosodic phrase boundaries (De Carvalho, Dautriche, Lin, & Christophe^78^; De Carvalho et al.^79^; Dautriche et al.^80^; Seidl & Cristià^81^; Soderstrom et al.^82^). The intonational contour of an O, S*ba*OV sentence is rising at the end of the topicalized object, which is consistent with the observations by Wang and Xu^22^, who found that discourse-initial topics are encoded with a raised pitch range and a sentence-final intonation generally has a falling intonation in Mandarin. That is, in spite of the pause, the prosodic properties of the experimental O, S*ba*OV items do not correspond to those of two utterances one after the other.

These prosodic properties of O, S*ba*OV appear to be language-specific and, if so, need to be learned by infants exposed to Mandarin. Alternatively, as an anonymous reviewer points out, an intonational contour in which fronted topics have increased salience due to pitch may be a universal feature (the topicalization structures OS(O)V of French explored in Lassotta et al.^17^ also feature a pitch rise on the topicalized object and a pause between object and subject); this potential universality would be worth investigating. There are no studies to date on different behavioral or neural responses to the marked prosody of this sentence type in infants, and so this remains for future research. For slightly older children, Chen^83^ investigated the production of topic-comment structures and found that the majority of the topic-comment structures produced by Mandarin-speaking children at ages 2;2 and 2;8 had an O, SV word order. Thus, from early on children acquiring Mandarin show sensitivity to the object’s presence and its role in OSV structures, and they are able to produce it. If the topic-comment structure is essential (given that Chinese is a topic-prominent language), it is no surprise that children’s language production is not guided by the agent-first parsing strategy, as almost any argument can occupy the topic position and, if our argument on the interpretation on O, S*ba*OV for our infants is on the right track, there would be continuity between the comprehension at an early age and production at 2;2. An alternative interpretation of our results, according to which infants ignore the initial O and parse the subsequent S*ba*OV sequence, would require that they ignore the intonational contour of the whole sequence, which includes O, and also fail to attach O to the sentence. As pointed out by an anonymous reviewer, under this alternative view, infants would be predicted to fail with S,*ba*OV; under our assumptions, infants would be expected to be able to parse sentences with topicalized subjects (both S,*ba*OV and subject left-dislocation structure S, VO); testing these subject topicalizations remains for future research.

Under the grammar-driven performance analysis, the behaviour of the infants with O, S*ba*OV indicates that infants in this age-range are able to parse sentences with non-canonical word orders in which the object has been moved to a sentence-initial position (Gertner and Fisher^84^ found that 21-month-olds mistakenly interpreted intransitive sentences with coordinated subjects as two-argument transitive sentences and assigned an agent role to the first of the two arguments. In our study, we did not find any similar effect, as infants did not interpret the sentence-initial object as agent; Franck et al.^62^ didn’t find that effect with SOV in French either.). This conclusion is in line with previous studies conducted in languages such as English (Seidl et al.^85^), French (Lassotta^18^ and Lassotta et al.^17^), and non−Indo−European languages like Japanese (Sano^86^; Shimada et al.^87^) and Korean (Shin^88^), in which infants show awareness of the displacement of constituents in the sentence. According to these studies, before the two−word stage infants have already acquired some grammatical knowledge related to syntactic movement (see also Perkins & Lidz^89^ on the correct interpretation of non−local syntactic dependencies between 17 and 18 months of age) (There is still another possibility: infants resort to partial knowledge of syntax, as proposed by Gagliardi et al.^90^, Perkins and Lidz^91^. In this view, infants resort to partial knowledge to interpret sentences involving fronted objects without having knowledge of the movement operation. According to Lidz et al.^92^, infants can recognise the absence of an argument in its canonical position and fill it in with the extraposed argument. However, this is only possible if infants have access to the argumental structure of the verb in the experimental items. In our study the verb used is a pseudo-verb, and therefore this third option can be ruled out.). In addition, the absence of an age effect in our results suggests that the parameter(s) related to the movement of the object have been fixed earlier than 17 months. Although the current study was not designed to probe the role of lexical knowledge in sentence interpretation, with the CDI data collected we were able to see that higher vocabulary scores failed to predict individual performance. That is, under the grammar−driven hypothesis, 17−month−old infants’ ability to deploy syntactic knowledge did not seem to depend on their productive vocabulary knowledge, in line with He and Lidz^93^.

Note that the *ba* construction is furthermore scarce in the input (only 3.1% in child-directed speech, Zhu & Gavarró^47^) and an analysis of the CHILDES corpus of child-directed speech showed that OS*ba*OV sentences are present by 1% (Yeh^49^). This means that, if our contention is correct, children get very little exposure to the construction and yet are able to parse it from infancy. This is also the case for the French OSV structure; according to Lassotta^18^, Clitic Left Dislocations are only found in 0.1% of all clauses in the French Lyon corpus (Demuth & Tremblay^94^) and, despite their very low frequency, these sentences were comprehended at 22 months, a result at odds with the prediction of frequency-based models (Yang^52,53^).

The performance of the adults follows the same pattern as the infants’ but the effects are stronger. Adult fixations on the target video are sustained longer in time and of higher intensity; in fact, the infants’ fixations on the target are above chance, but sometimes close to it, as noted by an anonymous reviewer. We argue that there is continuity between infants and adults, but there are differences in the strength of the signal. The O, S*ba*OV condition in experiments 1 and 2 revealed an unexpected result: infants identified the target video faster than adults (in the first window, instead of the second). Experiment 3, run only with adults, was a follow-up to experiment 2 in which a single change was introduced: the baseline was modified in the O, S*ba*OV condition; the new baseline was pragmatically more appropriate to the experimental sentences, as it placed the focus on the object in such a way that a sentence with a topicalized object was more appropriate (‘What happened to the bunny? The bunny, the duckling V-ed.’). This pragmatically adequate lead-in had the expected effect, since adults identified the target video from the first window in experiment 3. The difference in adult performance between experiments 2 and 3 witnesses to the impact of pragmatic well-formedness in language comprehension in adults. This indicates pragmatic sensitivity on the part of the adults, while infants appear to be mostly sensitive to grammatical-prosodic information. Exploring this difference remains for future research.

## Conclusion

We have presented the results of an experiment with infants exposed to Mandarin tapping on the comprehension of the canonical SVO word order and two non-canonical word orders involving the *ba* construction, S*ba*OV and O, S*ba*OV. The study was conducted in the preferential looking paradigm with the use of an eye-tracker, and we resorted to pseudo-verbs. The results show that infants with a mean age of 17.5 months directed their gaze to the video corresponding to the target event, rather than the theta-role reversal representation, from which we infer that these infants comprehend the sentences. We have considered two possibilities: either they are adhering to an agent-first parsing strategy, or they have built a grammatical representation attuned to the adult syntax. An agent-first parsing strategy is consistent with the results for SVO and S*ba*OV; this strategy is, however, more difficult to reconcile with the comprehension of the patient-first O, S*ba*OV sentences. We argue that an agent-first strategy cannot capture the infants’ target performance with the O, S*ba*OV experimental items, while a grammatical analysis, including its special left dislocation prosody, on the part of infants is more plausible. If so, our study provides the first evidence that Mandarin-learning 17.5-month-olds can assign the correct patient-agent-Verb interpretation to non-canonical OSV sentences with pseudo-verbs before they actually produce two-word utterances. Infants at 17.5 months are not only able to parse sentences with different word order patterns, but also are sensitive to the certain functional heads of their target language (as *ba* in our study) based on the primary linguistic data. This is possible from very early even for low frequency functional categories. We argue that the evidence provided lends support to the hypothesis that infants develop early abstract knowledge of word order and the movement options of the language they are acquiring.

## Data Availability

The data that support the findings of this study are available from the corresponding author upon reasonable request.
